# Subsequent somatic axis and bone tissue metabolism responses to a low-zinc diet with or without phytase inclusion in broiler chickens

**DOI:** 10.1371/journal.pone.0191964

**Published:** 2018-01-26

**Authors:** Siemowit Muszyński, Ewa Tomaszewska, Małgorzata Kwiecień, Piotr Dobrowolski, Agnieszka Tomczyk-Warunek

**Affiliations:** 1 Department of Physics, Faculty of Production Engineering, University of Life Sciences in Lublin, Lublin, Poland; 2 Department of Animal Physiology, Faculty of Veterinary Medicine, University of Life Sciences in Lublin, Lublin, Poland; 3 Department of Bromatology and Food Physiology, University of Life Sciences in Lublin, Lublin, Poland; 4 Department of Comparative Anatomy and Anthropology, Maria Curie-Skłodowska University, Lublin, Poland; University of Illinois, UNITED STATES

## Abstract

Zinc is required for normal bone development and cartilage formation. The purpose of this study was to assess the effect of with adding organic Zn (alone or phytase inclusion) at the reduced dose to growing male Ross 308 chickens on somatic axis and bone tissue metabolism. 200 one-day old broilers were divided into the negative control group fed diet without Zn or phytase inclusion, positive control group receiving Zn in the 100% of daily recommended dose from ZnO, and two experimental groups fed diet introduced Zn in 25% of daily recommendation as a glycine chelate (Zn-Gly) with or without phytase inclusion (500 FTU·kg^-1^). Supplemental organic Zn increased bone Zn and Mg content, serum IGF-1, growth hormone and leptin concentration. Additional phytase inclusion increased body weight gain, blood plasma Ca, Fe, Zn and osteocalcin concentration and tibia ash percentage when compared to the Zn-deprived control. Bone geometry, yield and ultimate strengths were enhanced in both organic Zn supplemented groups, and the overall mechanical strength parameters of bone were better in these groups than in the positive control group supplemented with standard dose of inorganic Zn. Also marked improvements in the thickness of articular and the growth plate cartilages as well as real bone volume and thickness of metaphyseal trabeculae were achieved in all broilers fed Zn-supplemented diet irrespective of phytase inclusion, however, the highest cancellous bone mass and the best trabecular structure were noted after ZnO supplementation. In concludion, although dietary organic Zn given to growing broilers in 25% of daily recommended dose improved general bone properties and mechanical strength, the obtained results do not allow to unambiguously state that organic Zn supplementation at this level, even after phytase inclusion, is sufficient for proper bone development.

## Introduction

Zinc (Zn) is an essential metal micronutrient with particular physiological functions in all animals [1]. Zinc is necessary for maintenance of structural and functional integrity of biological membranes, facilitation of gene expression and protein synthesis, enzyme structure and function, appetite regulation and good food utilization [2, 3]. Zinc is closely linked to bone metabolism [1, 4]. Especially, zinc in bone is very important during the stages of rapid growth as it plays a crucial role as a catalyst of many enzymes that affect bone development, formation and metabolism [4, 5]. It interacts with hormones involved in bone growth such as insulin-like growth factor 1, osteocalcin, testosterone and insulin [1].

Phytate is the primary storage compound of phosphorus in seeds. It has the capacity to strongly bind cationic minerals making them unavailable as nutritional factors [6–8] and the binding strength of minerals to phytate has been identified as the strongest to Zn^2+^ [9]. Further, maximum binding of elements by phytate occurs at pH 6, which is the pH of the duodenum, where generally absorption of mineral cations takes place [10].

Exogenous phytases represent a subgroup of phosphomonoesterases that are capable of initiating the dephosphorylation of phytate [11, 12]. Depending on the position of the carbon in the myo-inositol ring at which dephosphorylation is initiated phytases have been divided into 3-phytases, 5-phytases and 6-phytases [11]. Supplementary phytases differ in terms of pH activity profile, pH stability and pepsin tolerance [11]. A number of studies have shown that phytase supplementation improves bioavailability of trace elements including Zn [6, 13–16]. By increasing the availability of these micronutrients, phytases may be able to replace the mineral premix in diets for broilers, thus the amount of trace elements added can be reduced [8]. It has been suggested that the amount of supplemental zinc may be lowered in a maize-soybean meal diet when phytate phosphorus utilization is enhanced [17, 18]. It has been shown that bioavailability of Zn in low-Zn diets was improved by adding microbial phytase [19]. Thus, to maintain mineral balance, not the absolutely ingested amount of a trace element is important, but rather its bioavailable amount [13].

Currently, the amount of Zn in the diet for Ross 308 broilers amounts to 100 mg of Zn per 1 kg of the diet, irrespective of its content in the basal diet [20]. Although severe zinc deficiency is uncommon in avian populations, marginal deficiency is likely to be much more prevalent, with associations to immune system dysfunction or restricted physical development, including musculoskeletal system. Recently, studies of Bao et al. [21], Kwiecień et al. [22, 23] and Tomaszewska et al. [24] have shown that the use of Zn in the form of glycine compounds at doses lower than recommended do not cause a deterioration in the physical, chemical, mechanical and morphometric properties of long bones in broiler chickens. Due to increased bioavailability of Zn from organic sources compared to inorganic ones, it could be possible to provide zinc supplements below recommended standards without compromising the performance associated with leg problems in poultry [22, 24, 25].

There are no reports about the effect of an organic zinc on tibial biomechanical traits and the histological bone development in broilers fed a low-zinc diet with or without phytase inclusion. Additionally the microscopic or histologic analysis of bone tissue in broilers is limited [26]. Thus, the objective of the present study was to investigate the histological, mechanical and chemical traits of the tibia and selected hormones involved in general growth and bone development in male Ross 308 broilers given organic Zn in a form of Zn-Gly chelate at the reduced level of 25 mg**·**kg^–1^ of the diet with or without the phytase addition.

Since Zn plays the important role in the general growth and bone development, it is hypothesized that the supplementation of Zn in a more bioavailable form of glycine chelate might be sufficient for proper development of the skeletal system in broiler, even if it is added to the diet in 25% of the total daily recommended dose. Thus, to evaluate the subsequent somatic axis and bone tissue metabolism responses to low-zinc diet from organic source with or without phytase inclusion in broiler chickens, the present study was performed with a common diet providing Zn in inorganic form of ZnO in the amount of 100% of daily recommended dose as a positive control group.

## Materials and methods

All procedures used in the research were approved by the Local Ethics Committee for Animal Testing at the University of Life Sciences in Lublin, Poland (No. 37/2011 of 17 May 2011).

### Animal, breeding and experimental design

In the experiment, healthy male Ross 308 broiler chicks (one-day-old straight run, n = 200) after individual weighing were randomly allocated to four dietary treatments: control group fed without additional Zn supplementation in premix (the NC group), first experimental group fed lowered level of organic Zn as glycine chelate (the Zn25% group) in premix, the second experimental group fed lowered level of organic Zn as glycine chelate with phytase in premix (the Zn25%+phyt group) and the group receiving Zn in 100% of daily recommended dose from ZnO (the PC group). Each group consisted of 50 birds comprised of 5 replicates of 10 birds each. The animals were reared on straw mulch and kept under standard rearing conditions with air temperature set at the optimal level depending on age [24]. The chickens had appropriate feed supplied *ad libitum* throughout the starter (1–21 days), grower (22–35 days), and finisher (36–42 days) phases of the experiment (Table 1) and constant access to fresh water. The starter diet was in the form of crumble, grower and finisher in the pellet form. The birds’ daily body weight gains were recorded to evaluate the growth rate. At the end of the experiment, after the 42^nd^ day of life, 10 birds of each group were randomly selected (2 birds per replicate per group) for slaughter. They were stunned by mechanical methods and then decapitated.

**Table 1:** Composition and nutritive value of the experimental diets.

Ingredient (%)	Starter (1–21 day)	Grower (22–35 day)	Finisher (36–42 day)
Maize	24.44	40.00	40.00
Wheat	42.99	27.84	28.84
Soybean meal*	25.0	24.97	22.87
Soybean oil	2.50	3.69	3.98
Monocalcium phosphate	0.90	0.90	0.81
Limestone	1.40	1.13	1.09
Sodium bicarbonate	0.08	0.08	0.08
Sodium chloride	0.29	0.25	0.26
Premix (without Zn)	0.50^a^	0.50^b^	0.50^c^
Fat-protein concentrate**	1.00	-	1.00
DL-methionine 99%	0.30	0.23	0.23
L-lysine HCl	0.42	0.28	0.27
L-threonine 99%	0.18	0.13	0.07
Nutritional value of 1 kg mixture:			
Metabolizable energy, MJ·kg^−1^	12.7	13.1	13.2
^d^ Crude protein, %	21.7	20.2	19.6
^d^ Crude fibre, %	2.41	2.32	2.31
^d^ Crude fat, %	4.52	5.28	5.64
^d^ Lysine, %	1.28	1.14	1.10
^d^ Methionine + Cysteine, %	0.94	0.84	0.83
^d^ Total Ca, %	0.87	0.79	0.76
^d^ Total P, %	0.67	0.66	0.64
^d^ Phytate P, %	0.32	0.32	0.32
^e^ Bioavailable P, %	0.35	0.34	0.32
^e^ Total Ca/bioavailable P	1.91	1.94	2.00
^d^ Zn, mg			
Zn from plants in basal diet, mg	28.32	25.87	24.99
25 mg Zn-Gly	51.28	50.42	50.81
25 mg Zn-Gly+phytase	50.49	51.03	50.85
100 mg ZnO	128.72	128.04	128.55

^a^ The premix provided per 1 kg of starter: Mn 100 mg, I 1 mg, Fe 40 mg, Cu 16 mg, Se 0.15 mg, vit. A 15 000 IU, vit. D_3_ 5 000 IU, vit. E 75 mg, vit. K_3_ 4 mg, vit. B_1_ 3 mg, vit. B_2_ 8 mg, vit. B_6_ 5 mg, vit. B_12_ 0.016 mg, biotin 0.2 mg, folic acid 2 mg, nicotinic acid 60 mg, pantothenic acid 18 mg, choline 1 800 mg

^b^ The premix provided per 1 kg of grower: Mn 100 mg, I 1 mg, Fe 40 mg, Cu 16 mg, Se 0.15 mg, vit. A 12 000 IU, vit. D_3_ 5 000 IU, vit. E 50 mg, vit. K_3_ 3 mg, vit. B_1_ 2 mg, vit. B_2_ 6 mg, vit. B_6_ 4 mg, vit. B_12_ 0.016 μg, biotin 0.2 mg, folic acid 1.75 mg, nicotinic acid 60 mg, pantothenic acid 18 mg, choline 1 600 mg

^c^ The premix provided 1 per kg of finisher: Mn 100 mg, I 1 mg, Fe 40 mg, Cu 16 mg, Se 0.15 mg, vit. A 12 000 IU, vit. D_3_ 5 000 IU, vit. E 50 mg, vit. K_3_ 2 mg, vit. B_1_ 2 mg, vit. B_2_ 5 mg, vit. B_6_ 3 mg, vit. B_12_ 0.011 μg, biotin 0.05 mg, folic acid 1.5 mg, nicotinic acid 35 mg, pantothenic acid 18 mg, choline 1 600 mg

^d^ analyzed values

^e^ calculated values

* 46% crude protein in dry matter

** 1 kg of fat-protein concentrate contains: 39% crude protein, 2% crude fat, 10.8 MJ metabolizable energy

The basal diets (Table 1), prepared on the basis of maize and wheat as well as soybean meal, containing 28.32 mg·kg^-1^ (starter), 25.87 mg·kg^-1^ (grower) and 24.99 mg·kg^-1^ (finisher) of Zn from plants as the feed basis, were formulated to meet or exceed nutritional requirements for Ross 308 broilers [20]. The nutrient composition of the basal diet was analyzed using standard methods. Total phosphorus was determined colorimetrically [27], phytic phosphorus spectrophotometrically after ashing at 550°C [28]. The Zn and Ca content in feed samples was determined using the FAAS technique [27]. The ion-exchange chromatography was used to determine the composition of amino acid in the diet [23]. Cysteine and methionine were determined in a separate analysis. The assimilable lysine was determined based on the difference between total lysine and so-called residual lysine [23].

### Supplementation of Zn amino acid chelate and phytase

The amount of Zn in the diet was based on nutritional recommendations for Ross 308 broilers i.e. 100 mg·kg^-1^ of Zn irrespective of its content in the basal diet [20]. The negative control group (the NC group) was fed a basal diet supplemented with the premix, which did not provide Zn (0 mg·kg^-1^). The experimental groups (the Zn25% and the Zn25%+phyt groups) were fed the basal diet and the premix, which provided Zn in the organic form as a glycine chelate (Gly-Zn) in the amount of 25 mg·kg^-1^, which is equal to 25% of daily recommended dose for Ross 308 broiler [20]. In this study a Glystar Forte chelate (Arkop, Bukowno, Poland) containing 16% of Zn was used. An application of Gly-Zn chelate was in accordance with the EU Directive 1334/2003 [29]. Additionally, the diet of the Zn25%+phyt group was enriched with 500 FTU·kg^-1^ of 6-phytase produced by a genetically modified strain of *Aspergillus oryzae* (Ronozyme® HiPhos, DSM Nutritional Products, Mszczonów, Poland). The positive control group (the PC group) was fed a basal diet and the premix, which provided Zn in the inorganic form as ZnO in the amount of 100 mg·kg^-1^, which is equal to 100% of daily recommended dose [20].

### Bone collection and analysis

Immediately after slaughter, the tibiae from individual chickens were dissected, cleaned from the remnants of adherent tissues and subjected to basic measurements. The relative bone weight was calculated as a ratio of the bird’s body weight and bone weight. After the measurements, tibiae were wrapped individually in gauze soaked in saline and kept frozen at -25°C until further examination. In subsequent stages of analyses, the right tibia was subjected to strength tests, while the bone collected from the left side of the chicken was earmarked for geometric measurements and histomorphometric analysis.

### Bone geometric parameters

Horizontal (medial-lateral plane) and vertical (anterior-posterior plane) external and internal external diameters of the mid-diaphyseal cross-section were measured with a digital caliper. On the basis of these measurements, the following geometric properties were calculated: mean relative wall thickness, vertical wall thickness, cortical cross-sectional area, cortical index, vertical cortical index, midshaft volume, second (cross-sectional) moment of inertia and radius of gyration about medial-lateral axis [30].

### Bone mechanical properties

The mechanical properties were determined after 3-hour thawing at room temperature. The three-point bending test of bone mid-diaphysis was performed on a Zwick Z010 universal testing machine (Zwick/Roell, Ulm, Germany). Prior to the analysis, the bone was placed horizontally on two rounded support bars. The distance between the supports was set in each case at 40% of the total bone length. The load was applied in the anterior-posterior plane of bone with a displacement rate of 10 mm·min^-1^ until fracture [30]. The determined mechanical properties were: yield strength, ultimate strength, bending moment, elastic strain and ultimate strain [30, 31].

### Histomorphometric analysis

After the geometric measurements, the sample of proximal end of each tibia with no visible lesions and degenerative changes were subjected to histology. Sagittal sections containing epiphysis and metaphysis cartilages were taken from the middle of the lateral tibial condyle strictly adhering to the previously described method and equipment [32]. Five sections from each tibiae were cut from each individual broiler chicken with a microtome (Microm HM 360, Microm, Walldorf, Germany). Goldner’s trichrome staining was used to assess the morphology of the growth plate cartilage and the articular cartilage. Safranin O staining was employed to the visual assessment of Mankin Histological-Histochemical Grading System to characterize the articular cartilage. Microscopic bright-field images were collected using a camera-equipped confocal microscope Axiovert 200M (Carl Zeiss, Jena, Germany).

The analysis of the collected images was performed with the use of Olympus cellSens software (Olympus, Tokyo, Japan). The thickness of the main zones: reserve zone (the zone I), proliferation zone (the zone II), hypertrophy zone (the zone III) and ossification zone (the zone IV) was measured at four sites along the growth plate cartilage as described previously [33]. Similarly, the thickness the following zones of the articular cartilage was measured: horizontal zone (superficial surface, the zone I), transitional zone (the zone II) and radial zone (the zone III) [34]. A total number of 200 measurements of each zone per group was performed (10 birds, 5 sections, 4 sites).

The Picrosirius red (PSR) staining was used to evaluate the morphology of the articular cartilage by the evaluation the distribution of thin (immature) and thick (mature) collagen fibres [35, 36]. The sections stained with PSR were observed under a Leica DM 2500 microscope (Leica Microsystems, Wetzlar, Germany) equipped with filters providing circularly polarized illumination. Documentation of the images was performed by a high-resolution CDD camera (Leica Microsystems, Wetzlar, Germany). Maturity of collagen was estimated by calculating the red versus green ratio, where red indicates thick, more mature collagen fibres and green indicates the thinner, immature collagen fibres in PSR sections [37].

The morphometric parameters examined were: bone volume (BV), tissue volume (TV), relative bone volume (BV/TV), trabecular separation (Tb.Sp), trabecular thickness (Tb.Th), fractal dimension (Fd) and trabecular number (Tb.N) of the trabecular bone [35]. Trabecular morphometry was measured using the public domain ImageJ software (Wayne Rasband, National Institute of Mental Health, Bethesda, Maryland, USA).

### Zn, Mg, Cu, Fe, Ca, P and ash content in bone

After evaluating their mechanical properties, the tibiae were mineralized in a muffle furnace at 600°C to determine the percentage of bone ash [34]. The percentage of ash was determined relative to the wet bone weight. The contents of calcium, zinc, magnesium, copper and iron in bones were determined by ICP-AES spectrometer PS950 (Leeman Labs, New Hampshire, USA). Total phosphorus content in the samples was determined spectrophotometrically with Helios-α apparatus (Unicam Instruments, Cambridge, UK). The content of minerals in the bone was calculated as its content of these components in crude ash.

### Blood collection

Blood samples were collected using standard venipuncture from brachial vein. Chickens were fasted overnight for 12 h before blood collection. Blood samples were collected in 6 ml Vacutest tubes containing lithium heparin. The plasma was obtained by centrifugation of whole blood at 3500 rpm for 15 min in a laboratory centrifuge and stored in Eppendorf test tubes at -80°C before analysis. Blood samples were also collected in tubes without anticoagulant for serum hormones concentration. Blood samples were allowed to clot for 30 min at room temperature and then centrifuged at 3500 rpm for 15 min. The separated serum was stored in Eppendorf test tubes and frozen at -80°C until assayed [38].

### Blood plasma biochemical analyses

The plasma concentrations of Ca, P, Zn, Cu and Fe were determined colorimetrically using a Metrolab 2300 GL unit (Metrolab SA, Buenos Aires, Argentina) and sets of ready-made biochemical reagents kits (BioMaxima, Lublin, Poland; Hydrex Diagnostics, Warsaw, Poland) according to the manufacturer’s protocol [38]. The manufacturer's declared intra-assay coefficients of variation (CVs) for the method were < 3.6, <1.9 < 2.4, <2.6, < 4.5% for the Ca, P, Zn, Cu, and Fe determinations, respectively. All analysis procedures were verified with the use of multiparametric control plasma as well as control plasma of normal-level and high-level elements content (BioCal, BioNorm, BioPath, respectively, BioMaxima, Lublin, Poland) [38].

### Growth hormone and bone turnover markers measurement

Serum concentration of chicken growth hormone (GH), osteocalcin (OC), insulin-like growth factor 1 (IGF-1) and leptin were determined using Benchmark Plus microplate spectrophotometer (Bio-Rad Laboratories, Inc., Hercules, CA, USA). Enzyme-linked immunosorbent assay kits (ELISA; Uscn Life Science Inc., Wuhan, China) with a minimum detectable concentrations of 0.056 ng·ml^-1^, 0.67 pg·ml^-1^, 7.4 pg·ml^-1^, 14.8 pg·ml^-1^, respectively, were used.

### Statistical analysis

Data are presented as least-square means (LSMeans) with their corresponding standard errors (SEM) in figures or pooled standard error of the means (pooled SEM) in tables. The normal distribution of the variables was tested for using the Shapiro–Wilk test, equality of variance was examined with the Brown-Forsythe test. The differences among the means were tested with the One Way ANOVA with *post hoc* comparisons made with the Tukey’s HSD. For all analyses, P < 0.05 was considered significant. Data were analyzed using Statistica 12 software (StatSoft, Inc., Tulsa, USA).

## Results

There were no treatment effects on final body weight, mean daily weight gain and feed conversion ratio in the NC group and Zn supplemented group without phytase (the Zn25% group) while the phytase supplemented group (the Zn25%+phyt group) did not differ from the PC group (Table 2). Total feed intake was the same in all groups.

**Table 2:** The effect of the supplementation with organic Zn in 25% of recommended dose with or without phytase inclusion on final body weight and mean growing rate of 42-day-old broilers.

Group	n	Body weight, g		Mean daily growing rate, g	Total feed intake, g	Feed conversion ratio
		Initial body weight	Final body weight			
NC	10	44.1	2363^a^	55.2^a^	4220	1.79^b^
Zn25%	10	43.8	2359^a^	55.7^a^	4162	1.77^b^
Zn25%+phyt	10	43.6	2641^b^	61.1^b^	4127	1.64^a^
PC	10	44.0	2578^b^	60.2^b^	4216	1.65^a^
Pooled SEM		0.2	41	0.9	65	0.02
P-value		0. 843	<0.001	0.016	0.676	0.033

^a, b^–mean values in rows with different letters differ significantly at P<0.05; Data given are LSMeans; SEM–standard error of the mean

NC–the negative control group without received Zn in premix

Zn25%–the group received Zn in 25% of daily recommended dose from Zn-Gly

Zn25%+phyt–the group received Zn in 25% of daily recommended dose from Zn-Gly with phytase (500 FTU) inclusion

PC–the positive control group received Zn in 100% of daily recommended dose from ZnO

The length and weight of the tibia as well as the bone weight to body weight ratio were not influenced by the diet type; however, the bone weight to length ratio decreased in the Zn25% group compared to the PC and phytase supplemented group; also the statistically significant decrease in the NC group was observed when compared to the PC group (P<0.001, Table 3).

**Table 3:** Physical, mechanical and geometric properties of tibia obtained from 42-day-old broilers in control groups and supplemented with organic Zn in 25% of recommended dose with or without phytase inclusion.

Item		Group				Pooled SEM	P-value
		NC (n = 10)	Zn25% (n = 10)	Zn25%+phyt (n = 10)	PC (n = 10)		
		Bone general properties					
Bone weight, g		22.8	21.1	25.1	23.5	0.8	ns
Bone length, mm		111	112	111	111	1	ns
Bone weight/bone length, g·mm^-1^		0.199^a^^b^	0.184^a^	0.224^b^^c^	0.235^c^	0.007	<0.001
Bone weight/body weight, %		1.12	1.04	1.02	0.94	0.05	ns
		Bone geometrical properties					
Horizontal internal diameter, mm		4.67^a^	5.81^b^	5.91^b^	5.68^b^	0.17	<0.001
Horizontal external diameter, mm		8.32^a^	9.49^b^	10.47^c^	9.82^b^^c^	0.28	<0.001
Vertical internal diameter, mm		4.29^a^	4.90^b^	5.29^b^	4.89^b^	0.14	<0.001
Vertical external diameter, mm		6.32^a^	8.12^b^	9.14^c^	8.24^b^	0.29	<0.001
Cross section area, mm^2^		26.3^a^	38.0^b^	50.5^c^	42.3^b^^c^	2.7	<0.001
Mean relative wall thickness,		0.67	0.65	0.75	0.71	0.03	ns
Cortical index, %		38.9^a^	38.9^a^	42.9^b^	41.2^a^^b^	0.75	0.009
Vertical cortical index, %		32.1^a^	39.7^b^	42.1^b^	40.4^b^	1.3	<0.001
Vertical wall thickness, mm		1.01^a^	1.61^b^	1.92^c^	1.67^b^^c^	0.09	<0.001
Midshaft volume, cm^3^		1.17^a^	1.71^b^	2.22^c^	1.69^b^	0.12	<0.001
Moment of inertia, mm^4^		86.4^a^	214.2^b^	358.3^c^	256.7^b^	30.6	<0.001
Index of gyration, mm		1.81^a^	2.38^b^	2.63^c^	2.38^b^	0.09	<0.001
		Bone mechanical properties					
Yield strength, N		163^a^	357^c^	293^b^	197^a^	21	<0.001
Ultimate strength, N		240^a^	441^b^	464^b^	289^a^	25	<0.001
Elastic stress, MPa		68.7^b^	75.6^b^	44.3^a^	33.6^a^	4.8	<0.001
Ultimate stress, MPa		100.9^b^	93.5^b^	71.0^a^	51.1^a^	6.0	0.001
Bending moment, N·m		1.81^a^	3.99^c^	3.27^b^	1.97^a^	0.25	<0.001

^a, b, c^–mean values in rows with different letters differ significantly at P<0.05; Data given are LSMeans; SEM–standard error of the mean

The description of the groups as in Table 1.

The Zn supplementation (irrespective of its source) significantly altered all measured geometrical properties of tibia. The Zn groups showed the increase in mid-diaphasys diameters, both internal and external (P<0.001). The inclusion of phytase further increased tibia external vertical and horizontal diameters when compared to the Zn25% group. As a result, the largest cross-sectional area and midshaft volume were found in the Zn25%+phyt group, while the smallest one was in the NC group (P<0.001). The mean relative wall thickness did not differ among groups. The cortical index increased for the Zn25%+phyt group (P<0.005), although did not differ when compared to the PC group. The vertical cortical index, calculated in anterior-posterior plane, showed an increase for all Zn groups, regardless of the presence of phytase or Zn source, when compared with the NC control (P<0.001). Further, as a result of differences in diameters, both the moment of inertia and index of gyration significantly increased in Zn supplemented groups and the greatest values were observed in the Zn25%+phyt group (P<0.001).

Also tibia mechanical properties differed among groups (Table 3). The maximum value of yield strength was observed for the Zn25% group. A reduction was observed when the phytase was added to the diet, however, it was still significantly increased when compared to both control groups (P<0.001). No differences in ultimate strength were observed between the Zn25% and the Zn25%+phyt groups and these values were significantly increased when compared to both control groups (P<0.001). When the mechanical endurance of bones is analyzed in terms of material properties of mid-diaphyseal part of the bone, it can be seen that bones of birds from the PC and Zn25%+phyt groups were subjected to significantly lower stresses before fracture when compared both with the NC and the Zn25% groups (P<0.001 and P = 0.001, for elastic and ultimate stress, respectively). The highest values of bending moment were observed in the Zn25% group (P<0.001).

**Fig 1:**
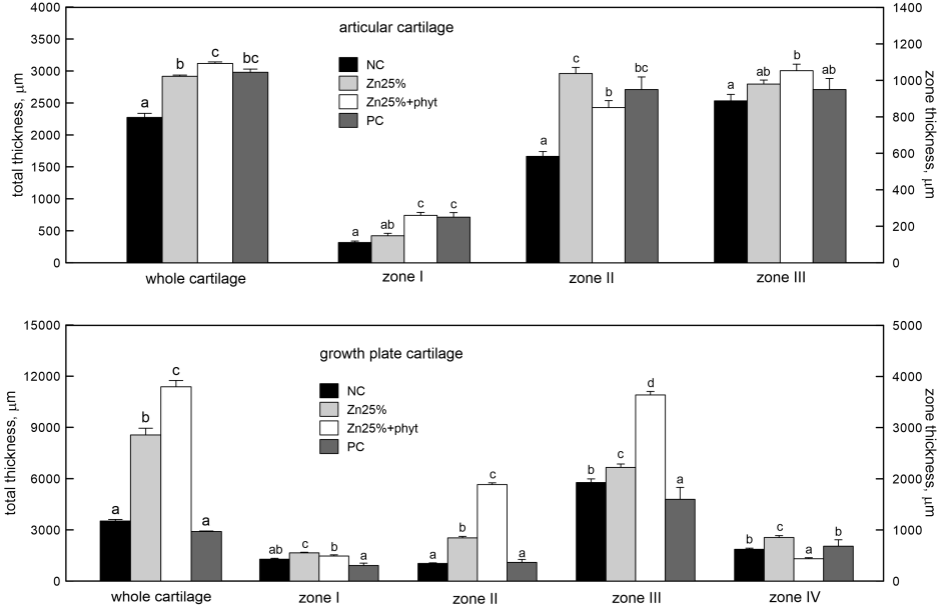
The morphology of articular cartilage and growth plate of tibia obtained from 42-day-old chickens in control groups and supplemented with organic Zn in 25% of recommended dose with or without phytase inclusion. Data given are LSMeans ± SEM; a, b, c, d–values with different letters differ significantly at P<0.05. NC–the negative control group without received Zn in premix. Zn25%–the group received Zn in 25% of daily recommended dose from Zn-Gly. Zn25%+phyt–the group received Zn in 25% of daily recommended dose from Zn-Gly with phytase (500 FTU) inclusion. PC–the positive control group received Zn in 100% of daily recommended dose from ZnO.

Proximal ends of the tibia of a birds fed without Zn supplementation were observed to have the thinnest articular cartilages among the all examined groups and the magnitude of increase of cartilage thickness was greater as phytase was added to Zn diet, although this did not differ from the thickness noted in the PC group (Fig 1A, P<0.001). This scheme applies to a thickness of all particular zones, except the zone II, where the thickest zone was observed for the Zn25% group instead of one supplemented additionally with phytase (P<0.001). The total thickness of the growth plate cartilage significantly increased when the organic Zn was added to basal diet and the increase in thickness was more than twofold in the Zn25% group and more than threefold in the Zn25%+phyt group when compared to both controls (Fig 1B, P<0.001). However, the zone I became wider only in the Zn25% group (P<0.01). The thickness of the zone II and zone III increased in both organic Zn groups and the magnitude of increase was greater as phytase was added to Zn diet (P<0.001). The thickness of the zone IV increased in the Zn25% group, while additional inclusion with phytase decreased the zone thickness when comparted to other groups (P<0.001).

**Fig 2:**
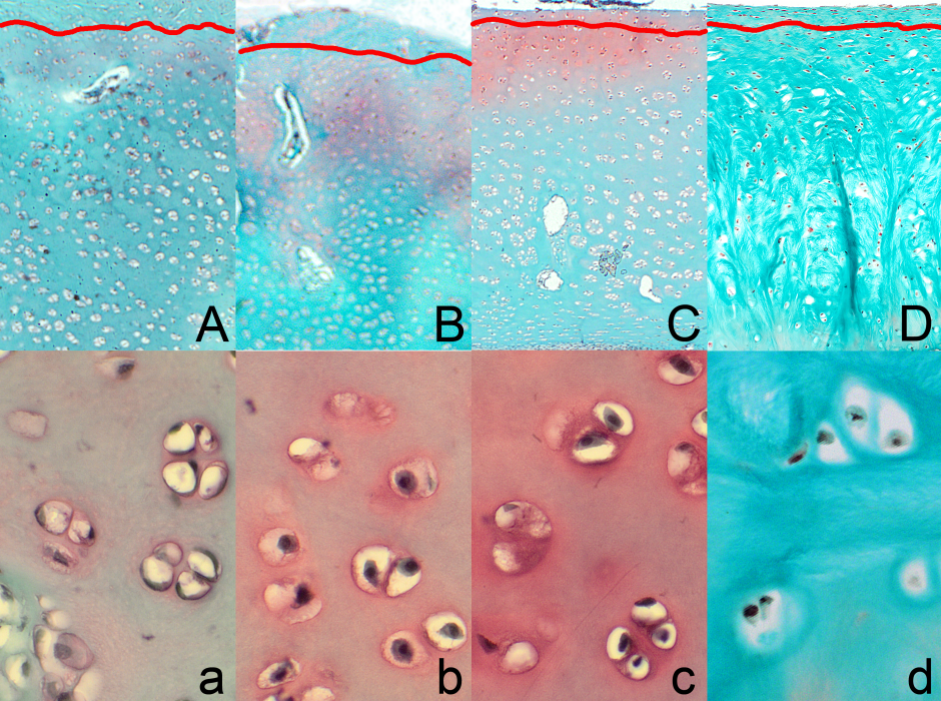
Representative images of safranin O staining carried out on formaldehyde-fixed sections from the tibial articular cartilage of 42-day-old chickens in control groups and supplemented with organic Zn in 25% of recommended dose with or without phytase inclusion. Vertical section of the zone I and upper part of the zone II of tibial articular cartilage from the NC group fed Zn-deficient diet (A), the Zn25% group (B), the Zn25%+phyt group (C), the PC group fed diet supplemented with ZnO in 100% of daily recommended dose (D). The description of the groups as in Fig 1. The red line shows the end of the zone I and the beginning of the zone II of the articular cartilage. Magnification x200. The cartilage from both control groups displayed very low proteoglycan content, displaying the weakest staining (a, d), while the chickens supplemented with Zn at the dose of 25% of daily demand demonstrated higher but not strong staining linked with higher content of proteoglycans (b). The most intensive staining with safranin O was observed around chondrocytes, especially in group with phytase inclusion (c).

Proteoglycan content in the articular cartilage was the lowest in both control groups, especially in the PC group. The supplementation of organic Zn enhanced their amount and the most intense staining pattern with safranine O indicating proteoglycans presence was observed in the zone II. Weak red color was in the extracellular matrix, strong red color was tightly around izogenous groups of chondrocytes in pericellular matrix. The most intense staining pattern was observed in the Zn25%+phyt group, especially evident in the zone I and beginning of the zone II (Fig 2C). More intensive red color was in extracellular matrix and the very strong intensive red color was present in pericellular matrix around izogenous groups of chondrocytes. Also the area of this staining was wider than observed in the Zn25% group (Fig 2B, Fig 2C).The surface of the articular cartilage of tibiae of birds from all examined groups was smooth without irregularities according to Mankin’s semiquantitative score system (Fig 2). Also the osteochondral junction was intact in all groups.

**Fig 3:**
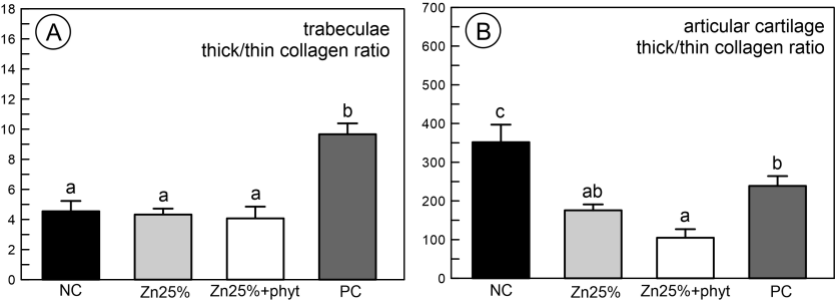
**Thick mature (red) to thin immature (green) collagen ratio in trabeculae (A) and articular cartilage (B) obtained from 42-day-old chickens in control groups and supplemented with organic Zn in 25% of recommended dose with or without phytase inclusion.** Data given are LSMeans ± SEM; a, b–values with different letters differ significantly at P<0.05. The description of the groups as in the Fig 1.

**Fig 4:**
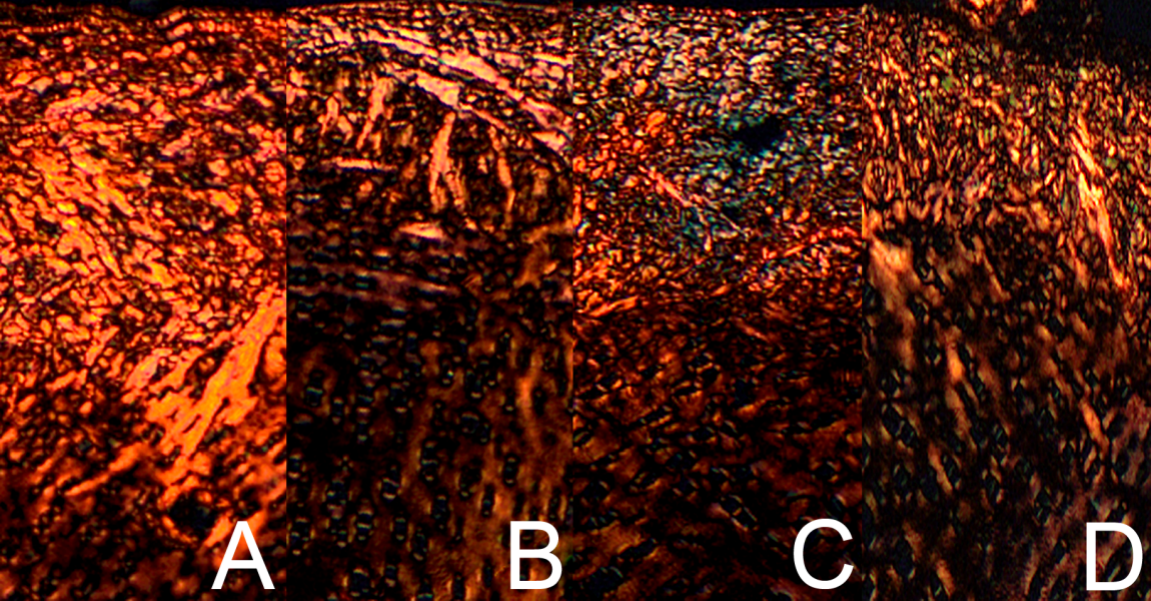
Representative images of PSR staining carried out on formaldehyde-fixed sections from the tibial articular cartilage of 42-day-old chickens in control groups and supplemented with organic Zn in 25% of recommended dose with or without phytase inclusion. Vertical section of the zone I of tibial articular cartilage from the NC group fed without Zn supplementation (A), the Zn25% group (B), the Zn25%+phyt group (C), the PC group fed diet supplemented with ZnO in 100% of daily recommended dose (D). The description of the groups as in the Fig 1. The large mature collagen fibres are orange or red and the thick ones including reticular fibres are green. Magnification x200.

The results obtained from the structural analysis of fibrous components in PSR-stained sections of bone revealed the difference in thick mature (red) to thin immature (green) collagen ratio in the trabeculae between PC and the other groups (Fig 3A, P<0.001). For the articular cartilage, the Zn supplementation resulted in decrease of collagen ratio, irrespective of the Zn source in premix, indicating a significant increase of the fraction of immature collagen fibres (Fig 3B, P<0.01). Fig 4 shows representative images of PSR staining carried out on formaldehyde-fixed sections from the tibial articular cartilage. The supplementation with organic Zn decreased thick (red) and enhanced thin (green) fibres in the articular cartilage, as greener radial fibres were observed (Fig 4B and Fig 4C). Furthermore, after phytase addition thin collagen fibres (green) were distinctly visible in the layer situated near calcified cartilage at the cartilage-bone interface (Fig 4C), compared to both control groups (Fig 4A and Fig 4D).

**Fig 5:**
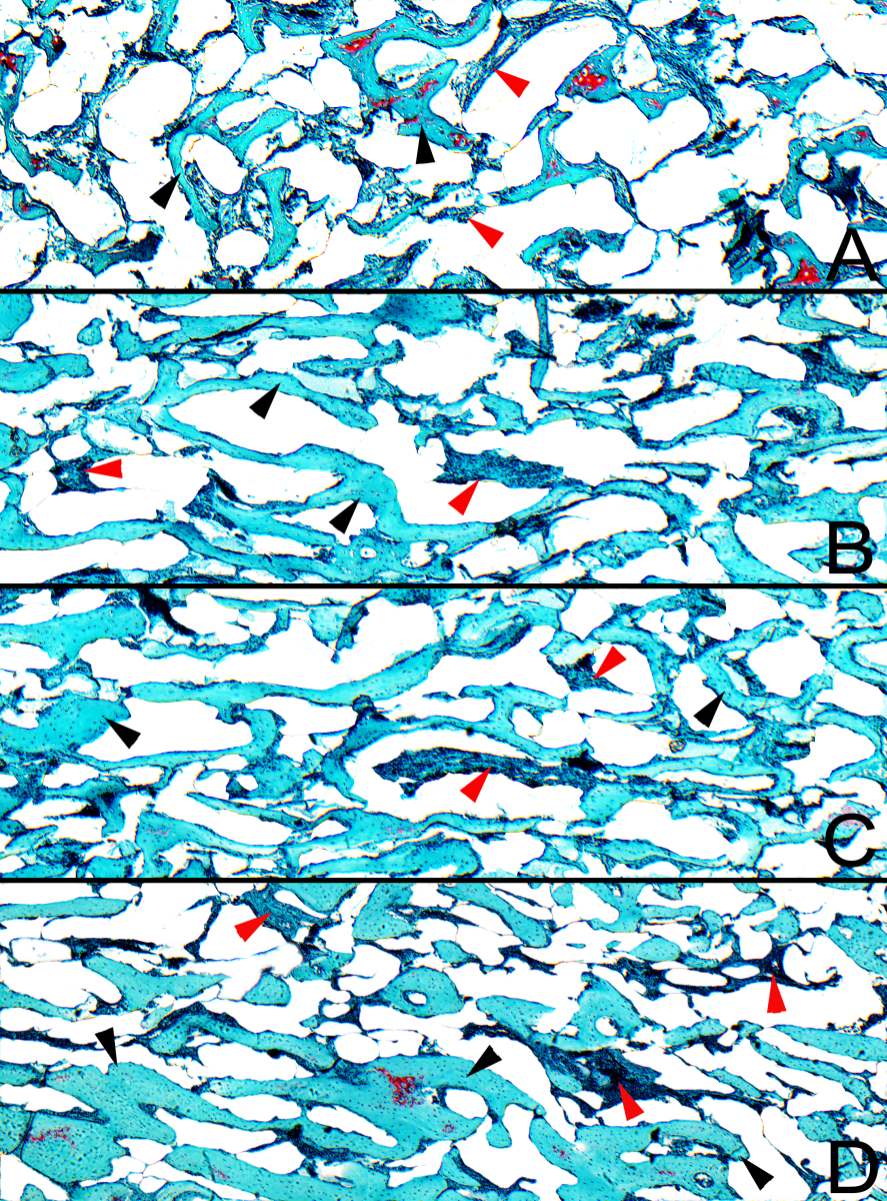
Representative images of Goldner’s trichrome staining carried out on formaldehyde-fixed sections from the tibial proximal part of 42-day-old chickens in control groups and supplemented with organic Zn in 25% of recommended dose with or without phytase inclusion. A–the NC group fed without Zn supplementation, B–the Zn25% group, C–the Zn25%+phyt group, the PC group fed diet supplemented with ZnO in 100% of daily recommended dose (D). The description of the groups as in the Fig 1. The real bone volume (BV/TV) and trabecular thickness (Tb.Th) significantly increased as Zn was added to the diet, with the highest value observed in PC group. A significant increase of trabecular space was additionally observed in the Zn25% group. The black arrow heads indicate trabecule, the red arrow heads indicate bone marrow. Magnification x50.

Histomorphometrical parameters of trabeculea of cancellous bone in tibia are presented in Table 4. The real bone volume (BV/TV) significantly increased in Zn supplemented groups, with the highest value observed in the PC group (P<0.05). Similar increase was observed for Tb.Th mean (P<0.001), while the both organic Zn diets did not affect the Tb.Th max value (Fig 5). A significant increase of one of the calculated indices of trabecular space, namely Tb.Sp max, was observed only in the Zn25% group (P<0.05). The organic Zn supplementation with or without phytase inclusion did not affect the fractal dimension Fd or the trabeculea number when compared to the NC group.

**Table 4:** Histomorphometrical parameters of trabeculea of cancellous bone in tibia obtained from 42-dy-old broilers in control groups and supplemented with organic Zn in 25% of recommended dose with or without phytase inclusion.

Item	Group				Pooled SEM	P-value
	NC (n = 10)	Zn25% (n = 10)	Zn25%+phyt (n = 10)	PC (n = 10)		
BV/TV, %	16.22^a^	20.27^b^	20.28^b^	27.00^c^	1.76	0.013
Tb.Th mean, μm	33.81^a^	41.65^b^	40.23^b^	100.98^c^	8.2	<0.001
Tb.Th max, μm	117.4^a^	144.0^a^	134.5^a^	215.5^b^	14.1	<0.001
Tb.Sp mean, μm	186.7	253.8	191.2	238.2	13.42	0.070
Tb.Sp max, μm	597.6^a^	731.5^b^	508.9^a^	469.4^a^	42.21	0.039
Fd,	1.542^b^	1.509^b^	1.549^b^	1.322^a^	0.024	<0.001
Tb.N, mm^-1^	4.75	5.09	5.07	2.69^a^	0.28	<0.001

^a, b, c^–mean values in rows with different letters differ significantly at P<0.05; Data given are LSMeans; SEM–standard error of the mean

BV/TV–relative bone volume; Tb.Th–trabecular thickness, Tb.Sp–trabecular separation, Fd–fractal dimension of trabecular bone; Tb.N–trabecular number

The description of the groups as in Table 1

**Fig 6:**
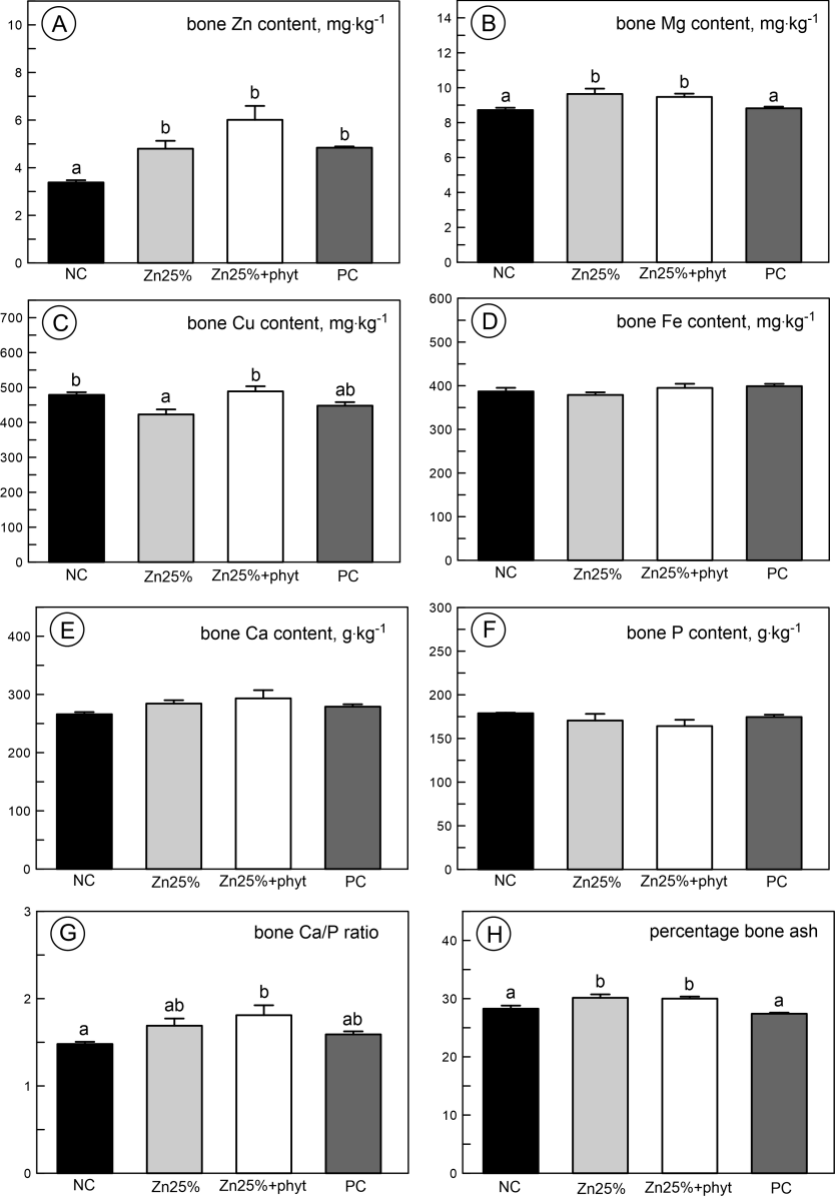
**The bone content of zinc (A), magnesium (B), copper (C), iron (Fe), calcium (E), phosphorus (F), bone Ca/P ratio (G), and crude ash (H) in 42-day-old chickens in control groups and supplemented with organic Zn in 25% of recommended dose with or without phytase inclusion.** Data given are LSMeans ± SE; a, b–values with different letters differ significantly at P<0.05. The description of the groups as in the Fig 1.

The Zn content in bone increased in all Zn supplemented groups (Fig 6A, P<0.01), while the Mg content in bone increased only in groups supplemented with organic Zn (Fig 6B, P<0.05). Cu content decreased in the Zn25% group (Fig 6C, P<0.01). Fe, Ca and P content in bone was not influenced by the diet type (Fig 6D, Fig 6E and Fig 6D, respectively), however the Ca/P ratio was the highest in the Zn25%+phyt group and bone ash content increased in groups supplemented with organic Zn (Fig 6G and Fig 6, respectively, P<0.05).

**Fig 7:**
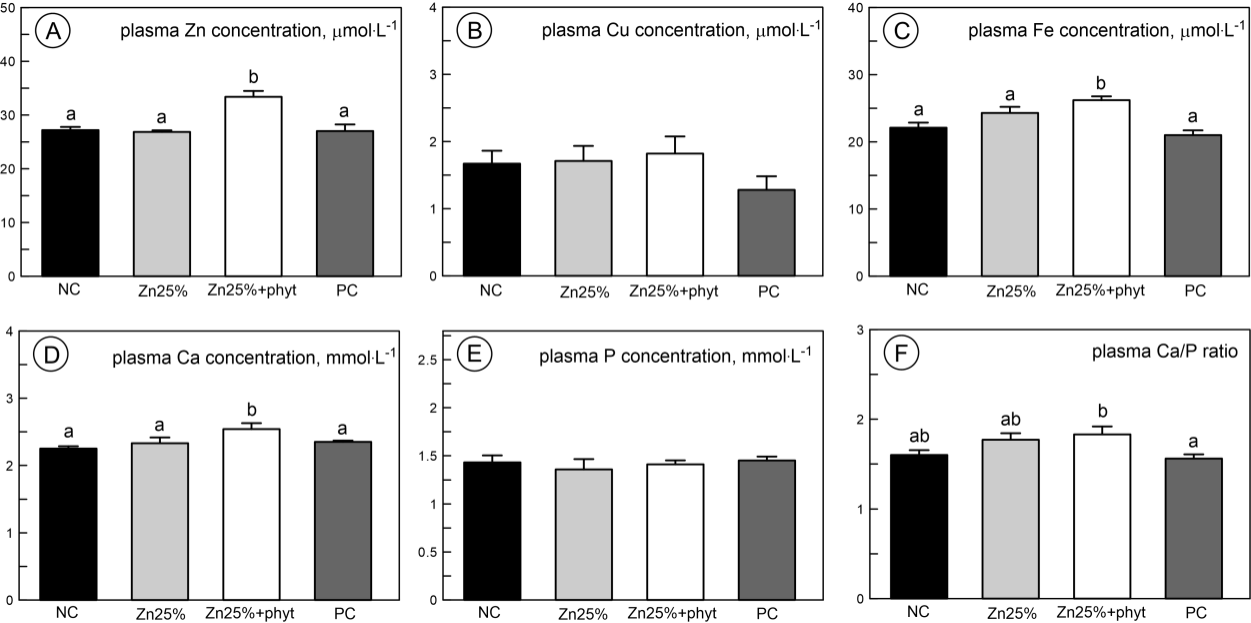
**The plasma concentration of zinc (A), copper (B), iron (C), calcium (D), phosphorus (E), and Ca/P ratio (F) in 42-day-old chickens in control groups and supplemented with organic Zn in 25% of recommended dose with or without phytase inclusion.** Data given are LSMeans ± SEM; a, b–values with different letters differ significantly at P<0.05. The description of the groups as in the Fig 1.

The plasma Zn, Fe and Ca concentration significantly increased when phytase was added (Fig 7A, Fig 7C, Fig 7D, respectively, P<0.01). The plasma P and Cu concentration was not significantly affected by Zn, irrespective of the Zn source or phytase inclusion (Fig 7C, Fig 7E, respectively). Consequently, the plasma Ca/P ratio was the highest in the Zn25%+phyt group (Fig 7D, P<0.05).

**Fig 8:**
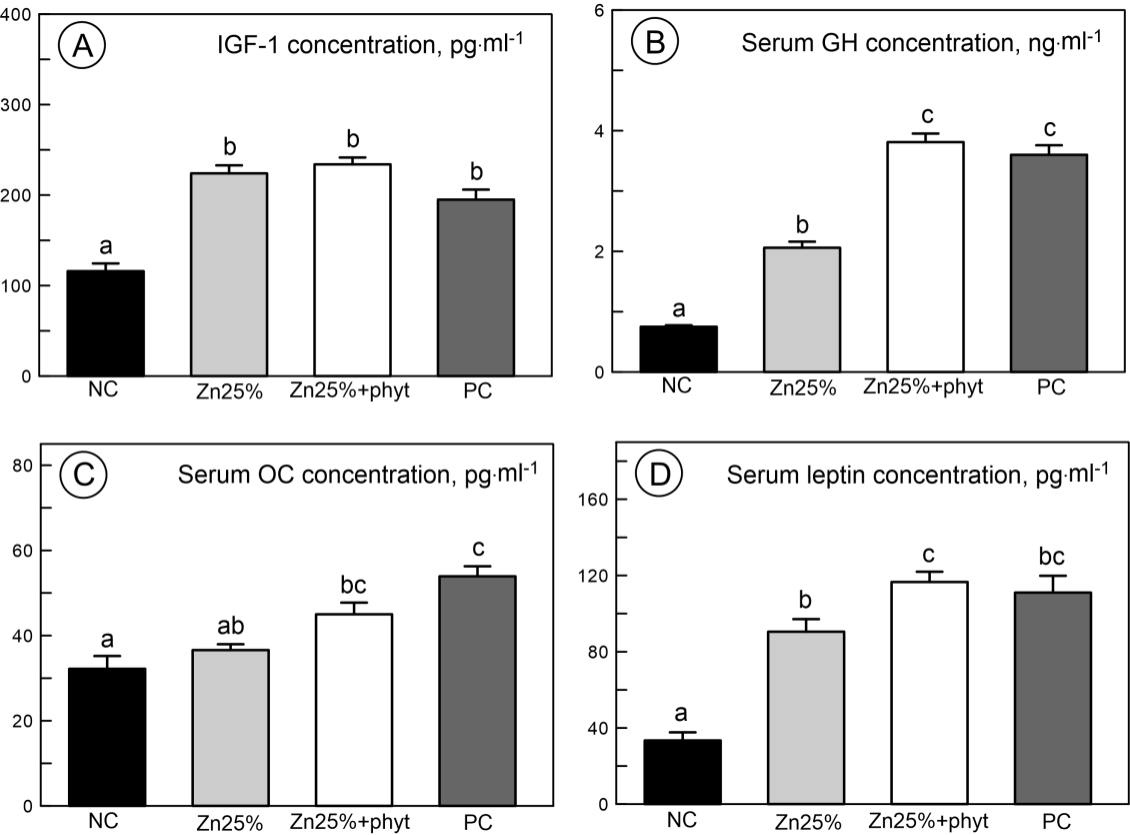
**The plasma concentration of insulin-like growth factor 1 IGF-1 (A), growth hormone GH (B), osteocalcin OC (C), and leptin (D) in 42-day-old chickens in control groups and supplemented with organic Zn in 25% of recommended dose with or without phytase inclusion.** Data given are LSMeans ± SEM; a, b, c–values with different letters differ significantly at P<0.05. The description of the groups as in the Fig 1.

The IGF-1 concentration significantly increased as the Zn was introduced into the diet (Fig 8A, P<0.001), but did not differ further when the phytase was added. Both serum growth hormone and leptin concentration significantly increased in Zn groups, with the magnitude of increase greater in the PC and the Zn25%+phyt groups than in the one with organic Zn administered alone (Fig 8B, Fig 8D, respectively, P<0.001). Zn supplementation did not alter the osteocalcin concentration but there was a significant increase after phytase inclusion to diet when compared to the NC group (Fig 8C).

## Discussion

For modern strains of boiler chicken one of the main problems associated with inappropriate or imbalanced nutrition is disorders in musculoskeletal system. These welfare problems occur because the rate of meat deposition in well-muscled and fast-growing breeds outruns the rate of development of the musculoskeletal system [39]. As skeletal system not only provides structural support for the bird but also is an important mineral source for metabolic needs, trace elements play a crucial role in the growth and development of skeleton. Zn-deficient diets have impairment in growth plate chondrocyte proliferation [40], insufficient mineralization, skeletal malformation [41, 42] and altered cartilage development [24]. However, researches concerning how the skeletal system will respond to low-Zn diet with and without dietary phytase are still limited and therefore required.

Our study showed that inorganic Zn given at the recommended dose in diet (100 mg·kg^-1^ of Zn as ZnO) resulted in one of the highest final body weights, body weight gains and the lowest feed conversion ratio. However, phytase inclusion to broilers supplemented with 25mg·kg^-1^ of Zn as Zn-Gly increased final body weight and growing rate as well as lowered feed conversion ratio at the same extent. These results are generally in agreement with several other studies where Zn supplementation at level of 15–25 mg·kg^-1^ did not influence body weight gain when compared to the Zn-deprived control [22, 43, 44]. On the contrary, some other studies show that low-Zn diet increases body weight gain when compared to Zn missing diets [14, 45–47], and, like it was shown in our study, this increase was even at the level comparable to standard diet [48]. Furthermore, in some studies dietary inclusion of phytase to Zn-supplemented diet does not influence the amount of ash in tibia [17, 19, 45], and this effect was also observed in our work. However, in birds supplemented with organic Zn percentage tibia ash was higher compared to both Zn-deprived and ZnO supplemented groups. In chickens, both growing rate and bone ash are sensitive indicators of bioavailability minerals [10] and above ambiguous results indicate that the effectss of addition of dietary phytase may depend not only on the level of supplementation but also on the phytase source or diet type [11].

Supplemental organic Zn at the level of 25% of daily recommendation significantly increased tibia yield strength, but this effect was dependent on the phytase inclusion in our diet and additionally followed from changes in geometric parameters. However, the increase of the ultimate strength was the same in both groups of chickens fed low-Zn diet irrespective of phytase inclusion. Our results agree with others, who report an influence of dietary Zn on the mechanical properties of bones [49–51]. On the other hand, there are studies showing that low-Zn diets do not increase bone breaking strength when compared to the Zn-deprived control [8, 21, 52] or group supplemented with ZnO at recommended dose [48]. But, as it was mentioned above, our broiler chickens fed low-Zn diet and phytase inclusion were one of the heaviest, therefore, both enhanced growing rate and greater load could influence the bone geometry, which did not differ from that of birds supplemented with inorganic Zn.

Thus, widespread bones in Zn supplemented groups suggest that Zn could improve bone formation. Our broilers fed low-Zn and phytase-included diet and those supplemented with ZnO had a significantly increased cross-sectional area and cortical index, indicating the higher bone wall-to-bone lumen ratio of mid-diaphyseal of their tibia. But, it should be emphasized once again that our broiler chickens fed low-Zn and phytase-included diet were heavier than others. It seems that it was an indirect effect of phytase supplementation, which resulted in the release of other minerals from phytate complexes. These additionally available minerals could play a significant role in the synthesis of hormones of somatotropic axis which also could be responsible for a faster growth observed in broilers from the Zn25%+phyt group. It agrees with other studies showing a better growth and bone development in poultry after added phytase to the diet, irrespective of analyzed mineral elements [26, 51, 53].

Furthermore, in our experiment bone Zn and Mg content significantly increased in both groups supplemented with 25 mg·kg^-1^ of organic Zn irrespective of phytase inclusion. On the other hand, the decrease of bone Cu content in the Zn25% group was observed, while in the group aditionally supplemented with the phytase this decrease was not noted. This is in agreement with another studies, as inclusion of Zn even at reduced dose generally increases bone Zn content, which in poultry is only dependent on the Zn concentration in feed [17, 21, 42, 43, 54]. Moreover, the fluctuations of the Zn level in blood serum can affect the actual Zn content in the bone ash. Also it is known that Zn exerts antagonistic effect on Cu absorption [10, 19]. Once again it should be mentioned, that the dietary inclusion of microbial phytase generally increases the bioavailability of several minerals, including Zn and Cu [8, 50]. The increased bioavailability of given element after phytase inclusion must not always result in the increased content of this element in bones, as, for example, in chicken only about 55% of total body Zn content is in bone and skeletal muscle [55]. Also no effect of low-Zn diet irrespective of phytase inclusion on bone Ca and P content was observed in our chickens. Probably, it was caused by the same progressive mineralization during growth and aging of our broiler chickens. It is in agreement with another study [21]. Thus, our results of the content of mineral elements in tibiae again could suggest that stronger bones in broilers fed low-Zn and phytase-included diet did not result from better mineralization of bone but improved bone geometry, probably following a better and more intensive general growth. However, the other factor could be involved in the improvement of bone mechanics e.g. bone microarchitecture.

Bone microarchitecture can also be an indicator of bone mechanical strength, independently of bone geometry or mineral density [56–58]. Zinc deficiency results in deterioration of the growth plate cartilage structure [40, 59], which leads to the decrease in cancellous bone mass (BV/TV) and to the deterioration of trabecular bone microarchitecture with thinner and less numerous trabeculae [40]. There is a study showing that rats fed a diet containing Zn at 25% of recommended dose maintain normal metaphyseal trabecularization [60], but it should be remembered that this study was performed on mammals. In the case of broilers, a recent study shows that the lowering of dietary dose of Zn in an organic form up to 25% of daily recommendation is insufficient to maintain proper bone microarchitecture compared to the control birds fed a reference diet [48], formulated according to requirements for Ross 308 broilers and providing Zn in an inorganic form [20]. The same result was obtained in presented study, as the highest cancellous bone mass and the best trabecular structure were noted in the tibiae of birds supplemented with ZnO at recommended dose (Fig 5). However, the same supplementation with organic Zn in both groups irrespective of phytase inclusion enhanced cancellous bone mass for about 25% compared to Zn-deprived control, mainly by increasing the thickness of trabeculae. But the changes in trabecular space were dependent on phytase inclusion, which presence led to its reduction. It is partially consistent with other observation [53]. Although when turkeys are fed a P-deficient diets, phytase inclusion improves trabecular bone architecture, bone Zn and Mg content [53]. Our previous study also proves, that phytase inclusion has no direct effect of trabecular architecture, as Cu supplementation in one fourth of recommended daily dose improves trabecular parameters irrespective of phytase in broiler chickens [58].

Furthermore, enhanced bioavailability of Zn or other trace elements can improve the morphology of bone cartilages. Zinc is also essential for bone formation process through a calcification in growth plate cartilage [26, 61]. It is suggested that growth plate cartilages of mineral-deficient avian are characterized by narrowing of the zones I and II and enlargement of the zone III [26, 53, 62, 63]. This was not found in our study when Zn and phytase were added. In our study, the widest zones II and III of the growth plate were observed in the Zn25%+phyt group. Thus, the observed lengthening of the growth plate zones cannot be associated with faulty osseous development, defective cartilaginous development or poor differentiation. The enlargement of the growth plate cartilage in Zn25% groups (irrespective of phytase inclusion) can be attributed to enlarged number of chondrocytes in the zone III and improved bone cell growth and maturation. The additional widening of the zone II of the growth plate cartilage after phytase inclusion is in agreement with other studies [26, 53].

From another point of view, Zn accumulated in bone tissues contributes to the differentiation of chondrocytes, fibroblasts and osteoblasts [1]. Osteoblasts are not only involved in bone mineralization and calcification but also participate in collagen synthesis [1, 5] and bone collagen content is raised in the presence of Zn [4]. Our study proves that Zn is essential for collagen synthesis, which is required for subsequent bone formation, as the highest trabeculae collagen ratio (Fig 3A) was observed in the PC group characterized by more abundant trabecular bone (Fig 5D). On the other hand, the presence of immature (thin) collagen in the articular cartilage observed in the Zn supplemented groups (Fig 3B) indicated that the bone turnover process was more intensive, especially in the 25%Zn+phyt group (Fig 4C). Furthermore, about 10% of different non-collagenous proteins, the expression of which is considered to be a specific marker, are expressed by osteoblasts and chondrocytes [64]. These non-collagenous proteins contribute to metabolic regulatory activities in an articular cartilage. Based on obtained results, one could conclude that Zn (irrespective of its form) and phytase inclusion in the diet improved the thickness of articular cartilage (Fig 1). However, the increased content of proteoglycans in the articular cartilage was observed only in broilers supplemented with organic form of Zn, irrespective of presence of phytase (Fig 2). Probably in that groups the elasticity of the articular cartilage was also improved, as proteoglycans have hydrodynamic functions [65] and their increased content could protect articular cartilage against the degradation by enabling it to withstand compressional forces in a fast-growing broiler chickens.

In our study, dietary supplementation of organic Zn at level of 25 mg·kg^-1^ without phytase inclusion had no effect on plasma Zn, Cu, Fe, Ca and P concentrations. It was consistent with the other studies also showing no differences in Zn, Cu, Fe and P concentration in blood plasma between groups supplemented with organic Zn-Gly at 25% of recommended dose and ZnO covering the whole recommended dose [22, 48]. On the other hand, the current results do not agree with several other findings where low-level supplementation with Zn from organic sources increases serum Zn, P or Ca concentration [14, 17, 19, 41–43, 61, 66]. There are also recent studies showing no effect of dietary Zn on plasma Zn and Cu concentration [44, 48, 67]. However, in the present study, Zn, Fe and Ca concentrations increased after phytase inclusion when compared to both control groups, irrespective of Zn supplementation. These findings are in accordance with previous reports on low-Zn dietary supplementation [17, 19]. Our data also agree with the other work where the serum P level is not affected by phytase [18].

May be the increase of the concentration of selected minerals (Zn, Fe, Ca) in blood plasma in our broiler chickens fed low-Zn diet and phytase inclusion triggered subsequent response of somatotropic axis. Zinc plays an important role in cell proliferation and differentiation by the stimulation growth hormone synthesis. It is involved in the production of osteocalcin and alkaline phosphatase, which provides calcium deposition in bone mid-diaphysis [1]. Zinc also has been found to influence serum leptin level [68] and stimulate the activation of IGF-1, a somatomedin which acts in response to the stimulation by growth hormone [4]. In our study, the presence of dietary Zn enhanced the level of growth hormone, IGF-I and leptin. The inclusion of phytase led to further increase in growth hormone and leptin concertation. This indicated that the positive effects of phytase inclusion in the low-Zn diet on growth and development were mediated by improved activity of the somatotropic axis, as bone and cartilage homeostasis is regulated by GH acting via IGF-1 stimulation of the proliferation [58]. However, the response of bone metabolism followed by the alteration in somatotropic axis on phytase inclusion in low-Zn diet is still unclear as there is a lack of understanding about the extent to which phytase improves bioavailability and digestibility of organic forms of Zn [69]. It should be further investigated.

## Conclusions

The results of our study indicate that feeding with a diet supplemented with lowered amount of organic Zn promotes the growth and development of the bone and hyaline cartilage and phytase inclusion may additionally intensify this effect. Such response is mediated by alteration in somatotropic axis. However, obtained results do not allow to state whether in the industrial breeding such a dietary strategy would allow to achieve better results than a standard dietary practice.
